# Gingival thickness threshold and probe visibility through soft tissue: a cross-sectional study

**DOI:** 10.1007/s00784-022-04483-0

**Published:** 2022-05-03

**Authors:** Dimitrios Kloukos, Eleni Kalimeri, George Koukos, Alexandra Stähli, Anton Sculean, Christos Katsaros

**Affiliations:** 1 Department of Orthodontics and Dentofacial Orthopedics, School of Dental Medicine, University of Bern, Freiburgstrasse 7, CH-3010 Bern, Switzerland; 2 Department of Orthodontics and Dentofacial Orthopedics, 251 Hellenic Air Force & VA General Hospital, Athens, Greece; 3 Department of Periodontology, 251 Hellenic Air Force & VA General Hospital, Athens, Greece; 4 Department of Periodontology, School of Dental Medicine, University of Bern, Bern, Switzerland

**Keywords:** Gingiva, Ultrasound, Phenotype

## Abstract

**Objectives:**

The aim was to retrieve the threshold of gingival thickness (GT), where the attribute of gingival translucency through probe visibility was altered.

**Methods:**

In 200 patients, the soft tissue thickness was evaluated at both central mandibular incisors using ultrasound quantification (USD). Additionally, probe visibility was determined using a standard periodontal probe (PB) (CPU 15 UNC, Hu-Friedy), inserted 1 mm deep into the gingival sulcus. Frequencies and relative frequencies were calculated. Repeatability analyses and receiver operating characteristics (ROC) were conducted to determine the USD cut-off point for probe visibility.

**Results:**

Regression model indicated that the probe was not visible at a thickness of 0.82 mm for the mandibular left central incisor (95% CIs 0.77, 0.86) and became visible at a thickness of 0.69 mm (95% CIs 0.65, 0.72). The respective values for the mandibular right central incisor were 0.82 mm (95% CIs 0.77, 0.87) and 0.70 mm (0.68, 0.74). ROC analysis confirmed the retrieved regression results by indicating the best fitting balance for specificity and sensitivity at a thickness of 0.8 mm for both mandibular incisors.

**Conclusions:**

In the frame of the current study, the data revealed that gingiva becomes non-transparent at a thickness of approximately 0.8 mm.

**Clinical relevance:**

Probe visibility at mandibular incisors for the discrimination between thin and thick soft tissues was correlated with a gingival thickness of 0.8 mm and a high repeatability.

## Introduction

Gingival phenotype has come into focus in various clinical and research fields as pink aesthetic plays an increasingly important role in the judgement of treatment success. Historically, two basic types of gingival architectures were proposed, the “flat” and “pronounced scalloped” gingival biotype [1]; later, Seibert [2] coined the term “scalloped-thin” and “flat-thick biotype” that were added by the “scalloped-thick” type [3]. In 2017, the “World Workshop on the classification of periodontal and peri-implant diseases and conditions” revisited the subject and described the gingival phenotype as the three-dimensional volume of the gingiva. This included gingival thickness (GT) and keratinized tissue width (KTW) as part of the “periodontal phenotype” which encompasses both the gingival phenotype and the thickness of the buccal bone plate [3–5]. The knowledge of the gingival phenotype is an important parameter in treatment planning, in the prediction of the clinical outcome, and in the decision-making process of various dental treatments, including implant placement, periodontal, and orthodontic treatment [6–11].

As the evaluation of GT is rather uncomplicated when assessing gingival phenotype, it is desirable to have a simple tool at hand in order to determine its size, and thus an objective evaluation of GT is of clinical relevance. Different methods have been proposed including transgingival probing with a needle or periodontal probe, ultrasound, or visibility check with a color-coded or periodontal probe [12]. We previously assessed these methods in terms of accuracy and reproducibility and concluded that both the transgingival measurement with a periodontal probe as well as the ultrasound device produced precise enough results for every-day practice [12]. The ultrasound method is based on the ultrasonic pulse-echo-principle where ultrasonic pulses at a frequency of 5 MHz are being transmitted through the sound permeable tissue (1.518 m/s) until hitting the surface of a hard tissue where they are being reflected. Nevertheless, an ultrasound device might not be available in every practice, and transgingival probing besides the fact that it is an invasive procedure also requires local anaesthesia which induces local tissue volume increase. Conversely, the evaluation of probe visibility is still considered a simple and frequently used method [13]. However, this method largely lies in the hands and the eyes of the examiner and might be prone to inherent errors while the reproducibility of this method was 85% between duplicate measurements [13].

With respect to GT, the question arises whether a cut-off level in mm can be defined discerning between thick and thin gingival phenotypes and whether probe visibility can be correlated to a value in mm. The literature is inconsistent; while most commonly the threshold between phenotypes is set at 1.0 mm [13], others define a thin phenotype as below 1.5 mm and a thick one as above 2.0 mm [14]. However, there is general agreement that probe visibility is an indicator of a thin gingival phenotype [15].

Since currently data about the association of probe visibility with a certain tissue thickness is lacking, the aim of the study was to investigate the tissue thickness at both central mandibular incisors using probe visibility testing and ultrasound measurements in order to define a cut-off point for visibility.

## Material and methods

### Study design

This is a cross-sectional study for which ethical approval was obtained from the Institution’s Ethics and Research Committee (076/7592/06.05.2015) of 251 Hellenic AirForce Hospital, Athens, Greece. The study was performed in accordance with the Declaration of Helsinki of 1975 and its revised version of Tokyo in 2004.

### Patient recruitment

For this study, 200 orthodontic patients before or during treatment at the Department of Orthodontics and Dentofacial Orthopedics, 251 Hellenic Air Force Hospital Athens, were consecutively enrolled. All patients or their parent/caregiver/legal guardian provided written informed consent before any measurements were conducted. Patients at any stage of orthodontic therapy and of all ages were eligible for the study if all anterior mandibular teeth were present. Exclusion criteria were (1) presence of crown restorations or fillings at the cervical part of the anterior mandibular teeth, (2) pregnant or breast-feeding women, (3) presence of clinical signs of gingival conditions/diseases resulting in swelling or color change, or presence of increased probing depth (e.g. > 3 mm), (4) presence of labial recession, (5) intake of medication with any known effect on the periodontal soft tissues, and (6) presence of congenital anomalies or dental structural disorders. This study was conducted in accordance with the guidelines of the Declaration of Helsinki.

### Clinical parameters

A trained periodontist (GK) assessed GT and probe visibility at the central mandibular incisors, mid-facially on the buccal aspect of each tooth of each patient with the following sequence:

- Firstly, GT was measured and recorded using an ultrasound device. by placing the upper contour of the transducer tip at the gingival margin. The needed time for the ultrasonic pulses to travel back and forth through the tissue determines the tissue thickness. The diameter of the transducer tip is 3 mm with a weight of 19 g. Measurements were recorded at a resolution of 0.1 mm. The mean value of 5 consecutive measurements was registered.
- Secondarily, probe visibility through the gingiva was evaluated using a standard periodontal probe^2^ inserted 1 mm deep into the gingival sulcus. This was a single-ended, color-coded probe, with #30 handling and black markings from 1 to 15 mm. The method does not directly quantify GT; the gingival thickness is classified based on the visibility of the tip i.e. as thin when it is visible, and thick when it is not. In this case, visibility or not of the probe was recorded as a dichotomous variable. All assessments were done under natural light without any method of magnification. No dental or operator light was used to illuminate the oral cavity, in order to avoid light scattering or interference with the gingival transparency.

### Intra-examiner repeatability

The intra-examiner repeatability of the clinician (GK) who performed all clinical examinations was analysed by examining and re-examining both the central mandibular incisors of 40 volunteers (80 measurements correspond to 20% of the sample) within 2 days, with both methods (USD and PB).

Bias between the repeated USD GT measurements was assessed with paired *t*-tests and the respective repeatability coefficient was calculated [16]. Agreement between repeated PB visibility assessments was evaluated via the kappa statistic.

### Statistical analysis

Descriptive statistics (mean, standard deviation, range) were obtained for age and USD GT measurements, while frequencies and relative frequencies were calculated for PB visibility. Descriptive statistics of USD by PB visibility were also obtained. Ordinary least squares (OLS) regression models were applied with USD as the dependent and with PB visibility as the independent variable. Receiver operating characteristics (ROC) analysis was conducted in order to determine the optimum USD cut-off point in classifying PB visibility, while the corresponding ROC curves were obtained. All statistical analyses were conducted using Stata 13.0/SE software.^3^

## Results

Two hundred participants contributing 400 measurements were analysed. The mean age was 16.92 years old (SD 7.24). The results of the paired *t*-tests for bias are reported in Table 1. The repeatability coefficient for the USD GT measurements for the mandibular left central incisor was found 0.30, while the respective coefficient for the mandibular right central incisor was found 0.18. The kappa statistic for the agreement between repeated PB visibility assessment was equivalent between the mandibular left and right central incisors (0.615; agreement 90%; *p*-value = 0.018).

**Table 1:** The results of the paired *t*-tests for bias in the repeated ultrasound gingival thickness measurements

Tooth	Bias (stand.er.)	*p*-value	95% confidence interval
Mandibular left central incisor	0.04 (0.05)	0.443	(− 0.07, 0.15)
Mandibular right central incisor	< 0.001 (0.09)	1.000	(− 0.07, 0.07)

**Fig. 1:**
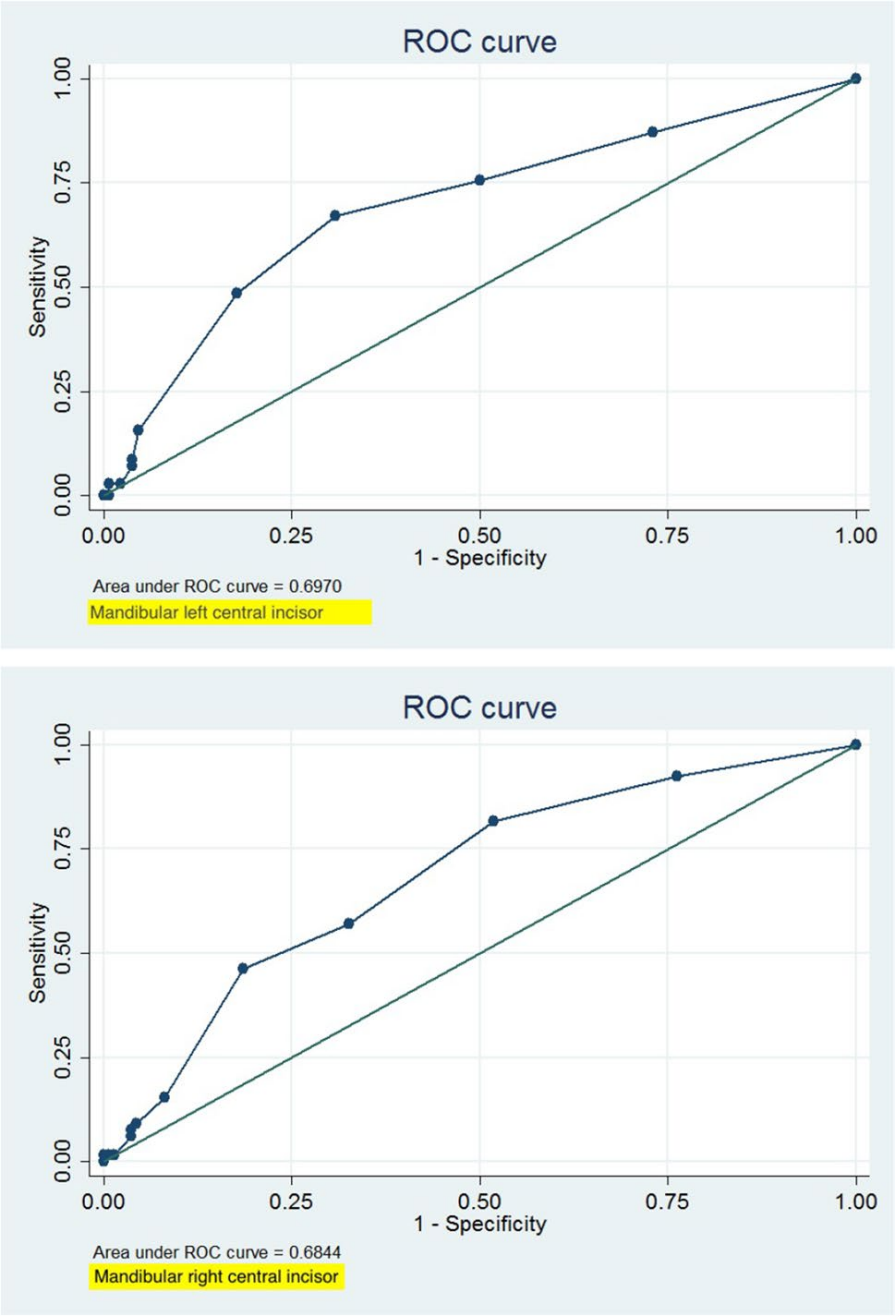
ROC curve for mandibular left and right central incisors

The descriptive statistics are reported in Tables 2 and 3, whereas the results of the OLS regression models are reported in Table 4. OLS regression model indicated that the probe was not visible at a thickness of 0.82 mm for the mandibular left central incisor (95% CIs 0.77, 0.86) and became visible at a thickness of 0.69 mm (95% CIs 0.65, 0.72) for the same tooth. The respective values for the mandibular right central incisor were 0.82 mm (95% CIs 0.77, 0.87) and 0.70 mm (0.68, 0.74). ROC analysis confirmed the retrieved regression results by indicating a balance for specificity and sensitivity at a thickness of 0.8 mm for both mandibular incisors. The ROC analysis results are displayed in Table 5 and the corresponding curves are illustrated in Fig. 1.

**Table 2:** Descriptive statistics for age and ultrasound gingival thickness measurements

Variable	Mean (SD)	Min	Max
Age	16.92 (7.24)	8	53
USD (mm)			
Mandibular left central incisor	0.73 (0.20)	0.5	1.5
Mandibular right central incisor	0.74 (0.21)	0.5	1.6

**Table 3:** Descriptive statistics of ultrasound gingival thickness measurements by probe visibility

Tooth	*N*	Mean (SD)	Min	Max
Mandibular left central incisor				
*PB visible*	130	0.69 (0.19)	0.5	1.5
*PB invisible*	70	0.82 (0.20)	0.5	1.4
Mandibular right central incisor				
*PB visible*	135	0.70 (0.20)	0.5	1.5
*PB invisible*	65	0.82 (0.21)	0.5	1.6

**Table 4:** Results of the ordinary least squares regression model. Bold values indicate statistically significant findings at the level of *α* = 0.05

	Coefficient (std.er.)	*p*-value	95% confidence interval
Mandibular left central incisor			
PB visibility			
*Not visible (Reference)*	0.82 (0.02)	** < 0.001**	(0.77, 0.86)
*Visible*	0.69 (0.02)	** < 0.001**	(0.65, 0.72)
*Difference*	− 0.13 (0.03)	** < 0.001**	(− 0.18, − 0.07)
Mandibular right central incisor			
PB visibility			
*Not visible (Reference)*	0.82 (0.02)	** < 0.001**	(0.77, 0.87)
*Visible*	0.70 (0.02)	** < 0.001**	(0.68, 0.74)
*Difference*	− 0.12 (0.03)	** < 0.001**	(− 0.18, − 0.06)

**Table 5:** Receiver operating characteristics (ROC) analysis. *AUC* area under the curve

Tooth	AUC (95% CI)	Cut-point	Sensitivity	Specificity	Correctly classified
*Lower left*	0.697 (0.619, 0.775)	≥ 0.5	100.00%	0.00%	35.00%
		≥ 0 0.6	87.14%	26.92%	48.00%
		≥ 0.7	75.71%	50.00%	59.00%
		≥ 0.8	67.14%	69.23%	68.50%
		≥ 0.9	48.57%	82.31%	70.50%
		≥ 1.0	15.71%	95.38%	67.50%
		≥ 1.1	8.57%	96.15%	65.50%
		≥ 1.2	7.14%	96.15%	65.00%
		≥ 1.3	2.86%	97.69%	64.50%
		≥ 1.4	2.86%	99.23%	65.50%
		≥ 1.5	0.00%	99.23%	64.50%
		≥ 1.5	0.00%	100.00%	65.00%
*Lower right*	0.684 (0.608, 0.761)	≥ 0.5	100.00%	0.00%	32.50%
		≥ 0 0.6	92.31%	23.70%	46.00%
		≥ 0.7	81.54%	48.15%	59.00%
		≥ 0.8	56.92%	67.41%	64.00%
		≥ 0.9	46.15%	81.48%	70.00%
		≥ 1.0	15.38%	91.85%	67.00%
		≥ 1.1	9.23%	95.56%	67.50%
		≥ 1.2	7.69%	96.30%	67.50%
		≥ 1.3	6.15%	96.30%	67.00%
		≥ 1.4	1.54%	98.52%	67.00%
		≥ 1.5	1.54%	99.26%	67.50%
		≥ 1.5	1.54%	100.00%	68.00%
		≥ 1.6	0.00%	100.00%	67.50%

## Discussion

According to the present results, gingiva translucency was associated with a gingival thickness of 0.7 mm and a high reproducibility. A non-visible probe was reported at 0.8 mm of GT. ROC analysis confirmed the balance between specificity and sensitivity at a thickness of 0.8 mm for both mandibular incisors. As in all diagnostic tests, sensitivity is a measure of how well the test can determine true positive results, and specificity is a measure of how well the test can determine true negative results. The association between specificity and sensitivity may have not been perfect in the case of the present study, which is easy to understand for any given comparison of methods; nevertheless, at the cut-off value of 0.8 mm, the best balance between these two properties was achieved. For all testing, both diagnostic and screening, there is usually a trade-off between sensitivity and specificity, such that higher sensitivities will mean lower specificities and vice versa. Thus, the goal of our interpretation was to balance this condition of trade-off between sensitivity and specificity.

The overall purpose of this cross-sectional study was to correlate gingival probe visibility as a simple test with the respective soft tissue thickness in mm. The focus was set on mandibular anterior teeth, since it is an area of great concern regarding aesthetic and functional considerations in relation to recession development, especially after a change of tooth inclination.

The issue of probe visibility through the gingiva and the respective correspondence to a certain or approximate GT has not been extensively discussed. Kan and coworkers [13] discerned thin and thick biotypes by three different methods and demonstrated that the probe visibility test was as accurate as direct measurements after tooth extraction. However, they did not correlate probe visibility with a value of GT. They suggested gingival biotype classification according to millimetres of gingival thickness with a cut-off level at 1.0 mm. According to them, GT below 1.0 mm was considered a “thin phenotype” and above 1.0 mm as “thick”. These measurements were conducted at maxillary anterior teeth. Others, however, found no correlation between probe visibility and GT, measuring again at maxillary incisors [17]. Our cut-off value for probe visibility is in line with Frost and coworkers [18] who studied 306 maxillary anterior teeth in 56 patients. They reported that in their sample probe invisibility was correlated with > 0.8 mm gingival thickness. However, they failed to identify a gingival thickness cut-off value for probe visibility.

Clinically, better treatment results of various dental procedures have been associated with thick gingiva. However, the question arises of how gingiva is classified as thick and thin in terms of millimetres. Gingival thickness < 1.5 mm was more likely to lose attachment after non-surgical periodontal therapy whereas values > 2 mm demonstrated no attachment loss [19]. In terms of complete root coverage, thresholds of > 0.8 mm and > 1.1 mm were proposed [20]. For guided tissue regeneration, post-treatment recession was more likely at sites with tissue thickness < 1 mm [21]. All aforementioned results point out that even slight differences may prevent an accurate identification of high-risk patients in regard to their soft tissue thickness; this, in turn, highlights the need for an accurate diagnosis.

Our results are in agreement with those of Baldi et al. [22] who have reported that a flap thickness > 0.8 mm (measured in the alveolar mucosa with a gauge) was associated with 100% of root coverage, thus pointing to a direct relation between flap thickness and amount of recession coverage. From a clinical point of view, our findings are extremely important since they provide support for the clinician in the decision-making process, especially on the need to use a soft tissue graft in conjunction with recession coverage surgery. Using a simple diagnostic method without the need for more sophisticated tools is confirmed in this large sample of participants.

When interpreting our results, it should be taken into account that the major strength of the current study, apart from the extensive sample size, is also the robust two-level analysis. On the other hand, limitations include the relatively young age of participants (mean age 16.92 years old, SD 7.24) and the absence of different ethnicities in the enrolled sample. Likely, the degree of gingival pigmentation might influence the ability to evaluate probe visibility. Further accessibility issues such as crowded or inclined teeth may have, as well, an influence on the clinical handling of the probe measurements as well as the visibility. Finally, only one examiner performed all measurements and therefore inter-examiner reproducibility was not included, which may be considered a methodological weakness.

Future research could focus on the use of ultrasonic imaging techniques with a transducer appropriate for gingival measurements. CBCT imaging and analysing may also be further investigated. From the periodontal point of view, the link between thin gingiva and risk of recession development, especially after orthodontic treatment, still remains to be prospectively evaluated.

In conclusion, within their limits, the present data revealed that gingiva becomes translucent at a thickness of approximately 0.7 mm. Discrimination between thin and thick soft tissues by evaluating probe visibility at mandibular incisors was correlated with a gingival thickness of 0.8 mm and a high repeatability.

## Data Availability

Data available on request from the authors (the data that support the findings of this study are available from the corresponding author upon request).

## Footnotes

2 PCP UNC15, Hu-Friedy, Chicago, IL.

3 StataCorp LP, College Station, TX, USA.
